# Response to hepatitis B virus vaccination in individuals with chronic hepatitis C virus infection

**DOI:** 10.1371/journal.pone.0237398

**Published:** 2020-08-26

**Authors:** Ashraf A. Ashhab, Holly Rodin, Marilia Campos, Ahmad Abu-Sulb, Jane A. Hall, Jesse Powell, Jose D. Debes

**Affiliations:** 1 Division of Digestive and Liver Diseases, Cedars-Sinai Medical Center, Los Angeles, California, United States of America; 2 Analytic Center of Excellence, Hennepin County Medical Center, Minneapolis, Minnesota, United States of America; 3 Department of Medicine, Gastroenterology and Hepatology, Hennepin County Medical Center, Minneapolis, Minnesota, United States of America; 4 Division of Pediatrics, Legacy Community Health, Houston, Texas, United States of America; 5 Department of Medicine, University of Minnesota, Minneapolis, Minnesota, United States of America; University of Cincinnati College of Medicine, UNITED STATES

## Abstract

**Background:**

Previous reports show conflicting results regarding hepatitis B virus (HBV) vaccine efficacy in Hepatitis C virus (HCV)-infected individuals.

**Aims:**

To evaluate HBV-vaccine response and identify possible factors that may contribute to lower vaccine efficacy in patients infected with HCV.

**Methods:**

We retrospectively evaluated all patients with chronic HCV infection at Hennepin County Medical Center, in Minneapolis, Minnesota, between 2002 and 2018. We addressed laboratory, liver-related, virus-related as well as vaccine-related variables, and their association to HBV vaccine response. Differences were tested using either a Chi-squared test or a T test to compare means between the two populations. Multivariate regression was modeled as a logistic regression.

**Results:**

1506 patients were evaluated, of which 525 received appropriate HBV vaccination and were assessed for response. Among those, 79% were vaccine responders and 21% were non-responders. On multivariate analysis, cirrhosis was associated with lower response to the vaccine (OR 0.6, CI 0.44–0.94). We found no significant differences for vaccine response in relation to smoking (87% vs 86%), IV drug abuse (74% vs 72%), Diabetes Mellitus (26% vs 22%) being on hemodialysis (2% vs.5%), or virus related variables.

**Conclusion:**

HCV infection seems to impair HBV vaccine response, with cirrhosis being the only identifiable risk factor for hypo-responsiveness among studied clinical and virus-related variables.

## Introduction

Hepatitis B virus (HBV) and hepatitis C virus (HCV) infections are the most common causes of chronic liver disease leading to cirrhosis and hepatocellular carcinoma worldwide. The world health organization estimates that in 2015, 257 million people were living with chronic HBV infection, and 71 million were living with chronic HCV [1,2].

Dual infection with HBV and HCV is not uncommon, as both viruses share some common paths of transmission and risk factors, including IV drug use, hemodialysis, and frequent blood transfusions such as in hemophilia [3].

Indeed, more than 25% of HCV-positive patients in the United States had positive markers for hepatitis B exposure, a proportion nearly six times that in the HCV-negative group [4]. Higher rates of cirrhosis and increased severity of liver disease have been reported with co-infection compared to either HBV or HCV mono-infection [5,6]. Additionally, HBV re-activation has been reported in HCV and HBV co-infected individuals receiving direct-acting antiviral (DAA) treatment, including individuals with either current or previous HBV infection [7,8]. Despite the availability of new DAA treatment for HCV and the realistic potential of HCV elimination, many HCV-treated patients continue to exhibit high risk behavior after treatment, putting them at risk for re-infection [9].

As such, HBV vaccine is recommended as the primary means to prevent HBV super-infection and its associated increase in morbidity and mortality in HCV-infected subjects [10].

In this regard, several reports suggest a similar HBV vaccine response rate among HCV infected individuals [11,12], while others suggest that this population mounts a poorer vaccine response [13,14]. This hypo-responsiveness is thought to be related to alterations in the cellular and humoral arms of the immune response secondary to HCV infection [15,16]. The impact of viral related variables such as viral load and viral genotype are controversial [13,17].

In light of the current availability of new DAAs which have effectively turned HCV infection into a curable disease, with well over 90% sustained virological response in most genotypes, attention should be focused towards HCV-related complications and indirect effects, such as poor vaccination responsiveness [18]. In this study we investigated the effect of HCV infection on HBV vaccination response, and the impact of clinical variables, life style, and virus related variables on vaccination response.

## Methods

### Study design and patients

This is a retrospective observational cohort study conducted through chart review of patients. Characteristics, clinical and virus-related variables of individuals infected with HCV who received HBV vaccination in Hennepin Healthcare, a county hospital in Midwestern US, between the years of 2002–2018 were reviewed.

For this study, 1506 electronic medical records (EMR) of individuals diagnosed with HCV (all available) were reviewed. The institutional review board of Hennepin healthcare approved the study. The data were analyzed anonymously therefore consent was not obtained from individual patients. The primary objective was to assess the response rate of patients with HCV to HBV vaccination, and the impact of clinical and virus related variables on response rate.

Clinical information of the patients were collected from EMR by the participating investigators. The data extracted included patients’ age at the time of Hepatitis B Surface Antibody (HBsAb) test, number of HBV vaccine doses received, potential risk factors (*ie*, smoking, IV drug use and alcohol use), presence of cirrhosis, Diabetes mellitus (DM), End Stage Renal Disease (ESRD) requiring dialysis, HCV viral load, and HCV genotypes. Patients with a positive hepatitis B core antibody were excluded from the study.

### Viral definitions

Patients were considered to have chronic HCV infection if they had a positive polymerase chain reaction assay (PCR) for HCV RNA at the time of receipt of HBV vaccination, and at the time of the HBsAb test check.

A positive HBsAb test was defined as having a titer of 12 mIU/mL or above. Patients were considered to be vaccine responsive if they developed a positive HBsAb test following at least one dose of HBV vaccine. Vaccine non-responders were defined as patients who did not develop a HBsAb following at least 3 vaccine doses. Complete vaccination was performed by injection of 20 μg recombinant HBsAg into the deltoid muscle at months 0, 1 and 6. Patients involved in the study received any of the following brands for HBV vaccination; Glaxo smith kline, Glaxo wellcome, Merck, Smith Beecham, Sanofi, Aventis, Abbot, and JHP. The majority of patients with known dates of the first vaccination dose received the first dose prior to HCV diagnosis (69%), however, most of the patients included in the study expressed a remote history of risk factors for HCV infection, suggesting early acquisition of HCV infection. Patients positive for Hepatitis B core antibody were removed from the analysis.

### Clinical definitions

Patients were considered to be cirrhotic at the time of the diagnosis if: a) they had a persistent International normalized ratio (INR) level of > 1.2, persistent high levels of total bilirubin (> 1.2 mg\dL) in addition to low platelets (< 150,000\mm) and radiographic cirrhotic morphology of the liver; b) cirrhotic liver morphology on imaging in addition to an INR of > 1.2, c) A combination of low platelet levels with high total bilirubin levels and an INR of > 1.2 at any time, d) liver biopsy or fibroscan indicating cirrhosis, or e) they had clinical stigmata of end stage liver disease (ESLD) [19]. Patients were considered to have ESRD if they had a glomerular filtration rate (GFR) of less than 15 mL/min/1.73 m^2^ and were receiving hemodialysis [20]. Patients were placed into a responsive group and a non-responsive group.

### Statistics

Statistical analyses were performed using SAS version 9.4. Differences between continuous variables were evaluated at the means using a T-Test. Categorical variables were evaluated using a Chi-Square test. The multivariate regression was modeled as a logistic regression.

## Results

### Demographics

We reviewed information of 1506 patients with chronic HCV infection. Of those, 85% percent (n = 1276) received HBV vaccination, 50% (n = 646) of which were checked with HBsAb test afterwards.

When comparing the patients who underwent HBsAb testing post vaccination to those who did not, both groups showed male predominance (70% vs 63%, respectively) and the group with post vaccination testing was slightly older, with mean ages of 54 (IQR 53–54) and 48 (IQR 47–50), p = 0.0001, in each groups respectively. Additionally, those who were not tested for HBsAb had lower rates of cirrhosis (14% vs 25%, p = 0.01).

Out of the 646 patients who were evaluated with HBsAb testing, 121 patients were excluded from the analysis as they were non-responders but had received less than 3 HBV vaccine doses.

**Fig 1:**
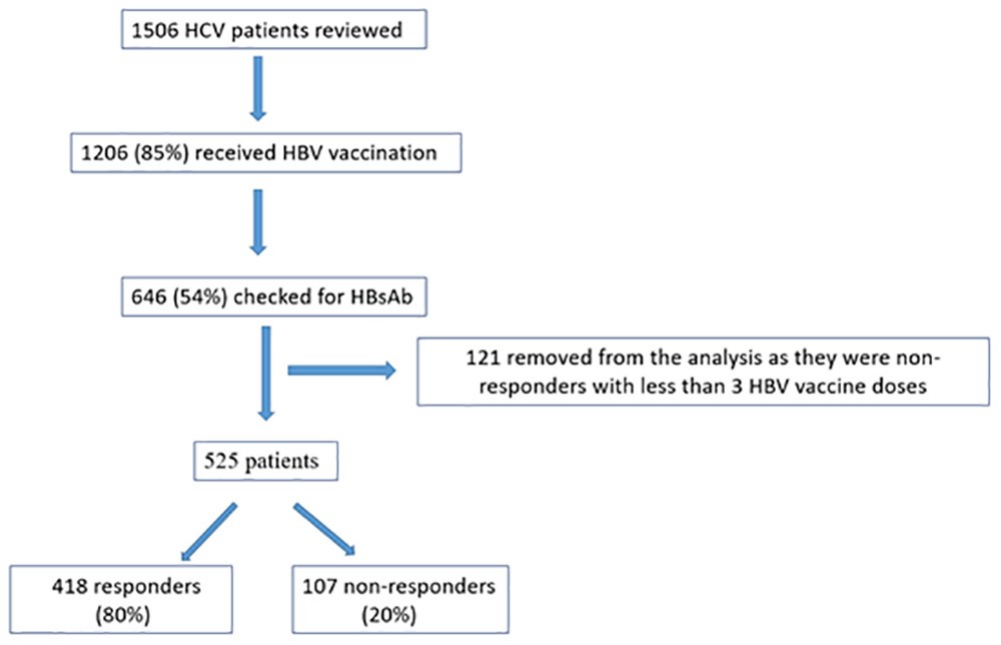
Vaccine responders and non-responders involved in the final analysis.

Among the remaining 525 patients, 80% (n = 418) developed HBsAb after vaccination with any number of HBV vaccine doses (vaccine responders) whereas 20% (n = 107) did not develop the antibody, following 3 or more HBV vaccine doses (vaccine non-responders) (Fig 1). Out of all the patients who received 3 or more doses in both groups, only 50% developed HBsAb.

When characterizing patients by the number of the HBV vaccines administered, 23% received 1 vaccine dose, 25% received 2 doses, 37% received 3, and 15% received over 3 HBV vaccines. Among the patients who received 2 doses, the response rate was 17%, compared to 42% in patients that received 3 doses (Table 1). The average number of vaccine doses in responders was 2.6, compared to 3.6 in non-responders (p<0.001) (Table 2).

**Table 1:** HCV patients’ response following various number of HBV vaccine doses.

Number of HBV vaccine doses	Proportion of HCV patients which were vaccinated (n = 413)	Proportion of responders (n = 185)	Proportion of non-responders to any number of vaccines (n = 228)
**1 dose**	23% (n = 95)	25% (n = 46)	21% (n = 49)
**2 doses**	25% (n = 103)	17% (n = 31)	32% (n = 72)
**3 doses**	37% (n = 153)	42% (n = 79)	32% (n = 74)
≥**4 doses**	15% (n = 62)	16% (n = 29)	15% (n = 33)

**Table 2:** Demographics, clinical and virus related-variables for responders with any number of doses vs non-responders.

	Responders (n = 418)	Non-responders n = 107	P value
**Average age at the time of HBsAB test**	52	51	0.6
**Males (%)**	69%	65%	0.4
**Females (%)**	31%	36%	0.4
**Average number of doses**	2.6	3.6	<0.001
**Presence of cirrhosis (%)**	24%	29%	0.4
**Alcohol use (%)**	79%	75%	0.4
**Tobacco use (%)**	87%	86%	0.8
**IV drug use (%)**	74%	72%	0.6
**Diabetes (%)**	26%	22%	0.4
**Dialysis (%)**	2%	5%	0.1
**Average HCV-RNA at HBsAb test check**	3246751	3586957	0.4
**Genotype 1a**			
**Genotype 1b**			
**Total genotype 1 (%)**	80%	79%	0.9
Genoype 3	10%	10%	
Other genotypes	10%	10%	

The demographics of the non-responder group were similar to the responders, with males comprising 65% and 69% of the cohort, respectively (p = 0.4). The median age at the time of the HBsAb test check was 57 years in non-responders, compared to 54 years in responders (IQR: 49–63 and 47–59, for non-responders and responders, respectively).

## Clinical variables

When comparing the clinical variables among the responders and the non-responders groups, we found no differences between the rates of alcohol use (79% vs 75%), IVDU (74% vs 72%) or Diabetes Mellitus (26% vs 22%), respectively. The proportion of smokers in both groups was similar (87% and 86% respectively). Only 5% of non-responders had end-stage renal disease and were on dialysis, compared to 2% of the responders (p = 0.1).

Notably, when assessing for the presence of cirrhosis, the rate of non-responders with cirrhosis was higher than that in the responders group (29% vs 24%), but the difference was not statistically significant (p = 0.4), (Table 2). However, on multivariate analyses that included age, gender, cirrhosis, alcohol abuse, and DM, only patients with liver cirrhosis were less likely to be reactive (OR 0.66, CI 0.44–0.94). When evaluating all cirrhotic patients who received 3 or more doses in the study, we found a response rate of 77% (n = 32/139).

A total of 393 HCV patients did not have cirrhosis in our cohort. Of those, 81% (n = 317) were responders (following any number of vaccine doses), whereas 19% (n = 76) were not (following at least 3 vaccine doses). When non-cirrhotics who received at least 3 vaccine doses in both arms were assessed, the response rate was found to be 51% (79/155).

### Virus-related variables

The average HCV viral load at the time of HBsAb test check among both of the groups was similar (3.5 x10^6^ vs 3.2x10^6^ IU/mL, for responders and non-responders respectively, p = 0.4). Both of the subgroups also had similar proportions of different HCV genotypes (Table 2).

Notably, all the above clinical and virus-related variables were also compared between responders who received 3 or more vaccine doses and non-responders, with the comparison yielding similar results (Table 3).

**Table 3:** Demographics, clinical and virus related-variables for responders who received ≥3 doses vs non-responders.

Variable	Responders (N = 107)	Non-responders (N = 107)	P value
**Average age at the time of HBsAb test**	51	51	1
**Males** (%)	67%	65%	0.7
**Females** (%)	33%	35%	0.7
**Average number of doses in those with ≧3 doses**	3.5	3.6	0.5
**Presence of cirrhosis** (%)	26%	29%	0.6
**Alcohol use** (%)	94%	75%	<0.0001
**Tobacco use** (%)	91%	86%	0.3
**IV drug use** (%)	72%	72%	0.6
**Diabetes** (%)	26%	22%	0.5
**Dialysis** (%)	2%	5%	0.3
**Average HCV-RNA at HBsAb check**	3220061	3586957	0.5
**Genotype 1a**			
**Genotype 1b**			
**Total genotype 1**	82	79	0.26
Genotype 3	13	11	
Other genotypes	5	10	

### Multivariate analysis

On multivariate analysis that included age, gender, cirrhosis, alcohol abuse, and DM, patients with liver cirrhosis were less likely to be re-active (OR 0.6, CI 0.44–0.94). Gender, age, alcohol abuse and DM did not show a significant correlation with HBV vaccine reactivity. These variables have been suggested before to correlate with incomplete response to vaccination [21,22].

## Discussion

To our knowledge, this is one of the largest studies to date to assess HBV vaccine responsiveness in HCV infected individuals, including both clinical and virus-related variables, and various numbers of vaccine doses. We found a significantly lower response to HBV vaccination in HCV-infected individuals, with an overall response rate of 79%, and a response rate of 50% when the cohort of those who received 3 or more vaccine doses was assessed. This is significantly lower when compared to the response rate of 90% to 98% reported in the general population [11,23]. Previous reports have found similar results, with sub-optimal to significantly lower response rates in HCV patients [24,25]. A rapid decline in the proportion of patients with protective levels of HBsAb over 1 year has also been reported in this population [26].

Interestingly, a number of studies found the HBV vaccine response rate to be similar regardless of HCV status [27,28]. In a recent meta-analysis of 11 studies involving 704 HCV patients, Liu et al concluded that chronic HCV infection can decrease the immune response to a standard schedule of hepatitis B vaccination [29]. Our study validates this finding with the advantage of having a similar number of patients within one study.

The cause for the lower vaccine response in HCV-infected individuals appears to be multifactorial. HCV patients who are HBV vaccine non-responders were found to have up-regulation of PD-1, a negative immune-modulator that correlates with diminished T cell activation in response to both general and virus-specific stimulation [15]. In a murine model, HCV core has been found to strongly inhibit the cytotoxic immune response [30,31]. Lastly, impairment in the humoral arm of the immune system has been noted in HCV patients and implicated in their poor HBV vaccine response [16]. Interestingly, some reports also suggest that HBV vaccine non-responders exhibit poor responses to tetanus toxoid and Candida as well [32].

Overall, the presence of these immune changes in HCV-infected individuals likely plays a large role in vaccine hypo-responsiveness, and the extent to which immune response is decreased could be a factor related to the discrepancy between some of the studies. Genetic variation could be also related to the discrepancy in vaccine response, with genetically determined low-responsiveness to HBsAg being reported in carriers of different HLA types such as B8, B44, DR3, DR7, and DQ2, however, this is less likely to be a major contributor to the response discrepancy among HCV patients [33,34].

In the vaccine non-responder population of our study there was a trend of no-response associated to the presence of cirrhosis, which was not significant on univariate analysis, but became evident on multivariate analysis. It has been reported that cirrhosis is associated with immune dysfunction involving both B-memory-cells, and T-cells, including helper T-cells, both of which are essential in mounting immunity to HBV vaccine [16,35]. Indeed, cirrhosis has been implicated in poor responsiveness to HBV vaccine in previous reports [36,37]. Similar to other studies, we found that clinical variables including age, gender, alcohol use, IV drug use and smoking were not significantly different between responders and non-responders [17,23]. DM was found to be a poor predictor for HBV vaccine response in patients with Chronic kidney disease (CKD) and was associated with lower response to both HAV and HBV vaccines when co-present with both HCV and fibrosis [38,39]. However, our study found similar rates of Diabetes Mellitus (DM) among responders and non-responders.

Virus-related variables such as HCV genotype and viral load have been shown to play a role in HBV vaccine responsiveness. One study showed a worse response to HBV vaccine in patients who had been infected by genotype-1 of HCV as compared to those infected by genotype 2 or 3, and some manuscripts have reported an inverse correlation between increased HCV viral load and response to HBV vaccine [18,22]. However, our study found no negative impact by HCV viral load or genotype on response to vaccination. This finding may be further suggested by reports noting no increase in HBV vaccine sero-conversion following HCV treatment with achievement of sustained virological response [26].

There is no current consensus regarding a differential HBV vaccination regimen in HCV patients. In our study, some vaccine non-responders seemed to develop sero-conversion with additional dose administration. Other vaccination regimens have been previously suggested in the literature. Monkari et al. achieved a higher response rate with double dose HBV vaccine in HCV patients compared to the standard dose, with 100% of their patients responding to 40 μgs administered at the standard 0, 1, 6 month regimen [40]. In the current age of DAAs however, re-vaccination for HBV post DAA treatment seems a sensible option to achieve maximum success.

There are several limitations to our study. Firstly, it is retrospective in nature. We did not characterize cirrhotic patients by MELD or Child-Pugh scores or diabetics by glycemic control, limiting our assessment of the impact in advanced stages of these disorders. Additionally, while a large proportion of our patients received over 3 vaccines to achieve sero-conversion, we did not assess the timeline during which extra doses were administered, and subsequently any possible variation in response rates based on early vs late administration.

In conclusion, we found that HCV infection significantly impairs HBV vaccine response, with liver cirrhosis being the main risk factor for vaccine hypo-responsiveness. We found no significant association of other clinical variables or virus-related variables with vaccine response rates. Prospective studies with HBV vaccination following DAA treatment are warranted.

## Supporting information

S1 File (XLSX)

## References

[1] Saravanan S, Velu V, Nandakumar S, et al Hepatitis B virus and hepatitis C virus dual infection among patients with chronic liver disease. J Microbiol Immunol Infect 2009;42:122–8. 19597643

[2] World Health Organization. (2017). Global hepatitis report 2017. World Health Organization. https://apps.who.int/iris/handle/10665/255016.

[3] Tyson GL, Kramer JR, Duan Z, Davila JA, Richardson PA, El-Serag HB. Prevalence and predictors of hepatitis B virus coinfection in a United States cohort of hepatitis C virus-infected patients. Hepatology 2013; 58: 538–45. 10.1002/hep.26400 23505059 PMC3729715

[4] Alter MJ, Kruszon-Moran D, Nainan OV, McQuillan GM, Gao F, Moyer LA, et al The prevalence of hepatitis C virus infection in the United States, 1988 through 1994. N Engl J Med. 1999;341:556–562. 10.1056/NEJM199908193410802 10451460

[5] Konstantinou D, Deutsch M. The spectrum of HBV/HCV coinfection: epidemiology, clinical characteristics, viralinteractions and management. Ann Gastroenterol. 2015;28:221–228. 25830779 PMC4367211

[6] Marot A, Belaid A, Orlent H, Sersté T, Michielsen P, Colle I, et al Characteristics of patients with hepatitis B virus and hepatitis C virus dual infection in a Western European country: Comparison with monoinfected patients. Clin Res Hepatol Gastroenterol. 2017;41:656–663. 10.1016/j.clinre.2017.05.003 28867077

[7] Belperio PS, Shahoumian TA, Mole LA, Backus LI. Evaluation of hepatitis B reactivation among 62,920 veterans treated with oral hepatitis C antivirals. Hepatology 2017; 66: 27–36. 10.1002/hep.29135 28240789

[8] Doi A, Sakamori R, Tahata Y, et al Frequency of, and factors associated with, hepatitis B virus reactivation in hepatitis C patients treated with all-oral direct-acting antivirals: analysis of a Japanese prospective cohort. Hepatol Res 2017; 47: 1438–44. 10.1111/hepr.12919 28585404

[9] Edlin BR, Carden MR. Injection drug users: the overlooked core of the hepatitis C epidemic, Clin Infect Dis, 2006, vol. 42 (pg. 673–676). 16447113 10.1086/499960 PMC1611492

[10] Centers for Disease Control and Prevention. Recommendations for prevention and control of hepatitis C virus (HCV) infection and HCV-related chronic disease. http://www.cdc.gov/mmwr/preview/mmwrhtml/00055154.htm. Accessed 25 February 2015.

[11] Khokhar N, Niazi TK, Qureshi MO. Effect of hepatitis B vaccination in patients with chronic hepatitis C. J Coll Physicians Surg Pak 2014; 24: 392–5. doi: 06.2014/JCPSP.392395 24953911

[12] Lee SD, Chan CY, Yu MI, Lu RH, Chang FY, Lo KJ. Hepatitis B vaccination in patients with chronic hepatitis C. J Med Virol 1999; 59: 463–8. 10534727

[13] Wiedmann M, Liebert UG, Oesen U, Porst H, Wiese M, Schroeder S, et al Decreased immunogenicity of recombinant hepatitis B vaccine in chronic hepatitis C. Hepatology. 2000; 31: 230–234. 10.1002/hep.510310134 10613751

[14] Lee SD, Chan CY, Yu MI, Lu RH, Chang FY, and Lo KJ. Hepatitis B vaccination in patients with chronic hepatitis C. J Med Virol. 1999; 59: 463–468. 10534727

[15] Moorman JP, et al Impaired hepatitis B vaccine responses during chronic hepatitis C infection: involvement of the PD-1 pathway in regulating CD4+ T cell responses. Vaccine. 2011;29:3169–3176. 10.1016/j.vaccine.2011.02.052 21376795 PMC3090659

[16] Doi H., Iyer T.K., Carpenter E., Li H., Chang K.M., Vonderheide R.H., et al Dysfunctional B-cell activation in cirrhosis resulting from hepatitis C infection associated with disappearance of CD27-positive B-cell population. Hepatology. 2012; 55: 709–719. 10.1002/hep.24689 21932384 PMC3245804

[17] Leroy V, Bourliere M, Durand M, Abergel A, Tran A, Baud M, et al The antibody response to hepatitis B virus vaccination is negatively influenced by the hepatitis C virus viral load in patients with chronic hepatitis C: a case-control study. Eur J Gastroenterol Hepatol. 2002;14:485–489. 10.1097/00042737-200205000-00004 11984145

[18] Forns X, Lee SS, Valdes J, et al Glecaprevir plus pibrentasvir for chronic hepatitis C virus genotype 1, 2, 4, 5, or 6 infection in adults with compensated cirrhosis (EXPEDITION-1): a single-arm, open-label, multicentre phase 3 trial. Lancet Infect Dis. 2017; 17: 1062–1068. 10.1016/S1473-3099(17)30496-6 28818546

[19] De Franchis R. Evolving consensus in portal hypertension. Report of the Baveno IV consensus workshop on methodology of diagnosis and therapy in portal hypertension. J Hepatol 2005; 43: 167–76. 10.1016/j.jhep.2005.05.009 15925423

[20] KDIGO 2012 Clinical Practice Guideline for the Evaluation and Management of Chronic Kidney Disease. 10.1038/ki.2013.243 23989362

[21] Schillie SF, Spradling PR, Murphy TV. Immune response of hepatitis B vaccine among persons with diabetes: a systematic review of the literature. Diabetes Care. 2012;35(12):2690–2697. 10.2337/dc12-0312 23173138 PMC3507602

[22] Yang S, Tian G, Cui Y, et al Factors influencing immunologic response to hepatitis B vaccine in adults. Sci Rep. 2016;6:27251 Published 2016 Jun 21. 10.1038/srep27251 27324884 PMC4914839

[23] Rahman F, Dahmen A, Herzog-Hauff S, Böcher WO, Galle PR, Löhr HF. Cellular and humoral immune responses induced by intradermal or intramuscular vaccination with the major hepatitis B surface antigen. Hepatology. 2000;31:521–527. 10.1002/hep.510310237 10655280

[24] Mattos AA, Gomes EB, Tovo CV, Alexandre CO, Remiao JO. Hepatitis B vaccine efficacy in patients with chronic liver disease by hepatitis C virus. Arq Gastroenterol 2004; 41: 180–4. 10.1590/s0004-28032004000300008 15678203

[25] Elefsiniotis IS, Vezali E, Kamposioras K, et al Immunogenicity of recombinant hepatitis B vaccine in treatment-naive and treatment-experienced chronic hepatitis C patients: the effect of pegylated interferon plus ribavirin treatment. World J Gastroenterol 2006; 12: 4420–4. 10.3748/wjg.v12.i27.4420 16865790 PMC4087759

[26] Chlabicz S, Grzeszczuk A, Lapinski TW. Hepatitis B vaccine immunogenicity in patients with chronic HCV infection at one year follow-up: the effect of interferon-alpha therapy. Med Sci Monit. 2002;8:CR379–383. 12011781

[27] Kallinowski B, Jilg W, Buchholz L, Stremmel W, Engler S. Immunogenicity of an accelerated vaccination regime with a combined hepatitis A/B vaccine in patients with chronic hepatitis C. Z Gastroenterol 2003; 41: 983–90. 10.1055/s-2003-42929 14562195

[28] Minakari M, Tahmasebi A, Motlagh MH, et al Efficacy of double dose recombinant hepatitis B vaccination in chronic hepatitis C patients, compared to standard dose vaccination. Int J Prev Med 2014; 5: 145–51. 24627739 PMC3950735

[29] Liu Jiaye, Wu Hui, Chen Hui. Immune response to hepatitis B vaccine in patients with chronic hepatitis C infection: A systematic review and metaanalysis. Hepatology Research 2018; 48: 119–126. 10.1111/hepr.13008 29197147

[30] Large MK, Kittlesen DJ, Hahn YS. Suppression of host immune response by the core protein of hepatitis C virus: possible implications for hepatitis C virus persistence. J Immunol 1999; 162:931–938. 9916717

[31] Bauer T, Jilg W. Hepatitis B surface antigen-specific T and B cell memory in individuals who had lost protective antibodies after hepatitis B vaccination. Vaccine. 2006 1 30;24(5):572–7. 10.1016/j.vaccine.2005.08.058 16171909

[32] Salazar M, Deulofeut H, Granja C, Deulofeut R, Yunis DE, Marcus-Bagley D, et al Normal HBsAg presentation and T-cell defect in the immune response of nonresponders. Immunogenetics. 1995;41(6):366–74. 10.1007/BF00163994 7759133

[33] Kruskall MS, Alper CA, Awdeh Z, Yunis EJ, Marcus-Bagley D. The immune response to hepatitis B vaccine in humans: Inheritance pattern in families. J Exp Med 1992;175:495–502. 10.1084/jem.175.2.495 1531063 PMC2119114

[34] McDermott AB, Zuckerman JN, Sabin CA, Marsh SGE, Madrigal JA. Contribution of human leukocyte antigens to the antibody response to hepatitis B vaccination. Tissue Antigens 1997;50:8–14. 10.1111/j.1399-0039.1997.tb02827.x 9243749

[35] Morita K., Fukuda Y., Nakano I., Katano Y., and Hayakawa T. Peripheral lymphocyte subsets vary with stage of hepatitis C virus-associated liver disease. Hepatogastroenterology. 2005; 52: 1803–1808. 16334781

[36] Tsai IJ, Chang MH, Chen HL, et al Immunogenicity and reactogenicity of the combined hepatitis A and B vaccine in young adults. Vaccine. 2000;19:437–441. 10.1016/s0264-410x(00)00205-x 11027806

[37] Keeffe EB. Acute hepatitis A and B in patients with chronic liver disease: prevention through vaccination. Am J Med. 2005;118(Suppl 10A):21–27. 10.1016/j.amjmed.2005.07.013 16271537

[38] DaRoza G, Loewen A, Djurdjev O, et al Stage of chronic kidney disease predicts seroconversion after hepatitis B immunization: earlier is better. Am J KidneyDis. 2003;42:1184–1192. 10.1053/j.ajkd.2003.08.019 14655190

[39] Chow KM, Law MC, Leung CB, Szeto CC, Li PK. Antibody response to hepatitis B vaccine in end-stage renal disease patients. Nephron Clin Pract. 2006;103:c89–c93. 10.1159/000092016 16534237

[40] Minakari M, Tahmasebi A, Motlagh MH, Ataei B, Yaran M, Kalantari H, et al Efficacy of double dose recombinant hepatitis B vaccination in chronic hepatitis C patients, compared to standard dose vaccination. Int J Prev Med. 2014;5:145–51. 24627739 PMC3950735

