# Supplementary material for: Response to hepatitis B virus vaccination in individuals with chronic hepatitis C virus infection
Source: PLoS One. 2020 Aug 26;15(8):e0237398. doi: 10.1371/journal.pone.0237398 (PMC7449383; doi:10.1371/journal.pone.0237398)
Supplement: Attachment — Submitted filename: Response to Reviewers.docx [file pone.0237398.s002.docx]

The authors appreciate the reviewers’ thoughtful and insightful comments, as well as the time and effort spent. We have changed the manuscript accordingly, and revised several of the analyses per the reviewers’ requests. Below, we provide responses to each comment (please note that page and line refer to the marked-up version).

**Reviewer #1:**

1. Comment: “Page 5: The sentence regarding vaccination with Euvax B is very confusing. This vaccine is not licensed in the U.S. and the next sentence describes other vaccine products used.”

**Response: The comment about Euvax has been removed, page 6, line 127.**

2. Comment: “I disagree with defining vaccine non-responders as those that received at least 1 dose. While this may be the definition in an intent to treat trial, we know from prospective registration trials that the rate of response from those who received only 1 dose is quite low and 2 doses is suboptimal. In the original registration trials from Szumness, the post-vaccine response (defined as >10 mIU/ml) was around 30% and this went up to around 70% after the second dose. In prospective trials the testing was done at short time intervals while your testing was randomly done, also decreasing apparent outcomes. At the minimum your study should focus on those who completed a full series.”

**Response: We have updated our definition of non-responders to that of those receiving 3 or more doses of the vaccine. We have updated all the comparisons and tables accordingly. That has led us to remove some of the data (such as average number of vaccine doses in non-responders) and to re-calculate some analyses, however, for the most part, the results of the study remained the same. Table 2 now contains comparisons between all responders to non-responders (page 19), and table 3 (page 20) now contains comparisons between responders with 3 or more doses, to non-responders. Any previous data related to comparisons involving non-responders with less than 3 vaccine doses were removed from the manuscript. We also added results from Multivariate analysis, again, with non-responders being modified to only those who received 3 or more doses (page 9, lines 204-207).**

3. Comment “Page 6: Your definitions of cirrhosis are very non-specific. Why not apply FIB-4 which is imperfect but is at least standardized in HCV positive patients and call cirrhosis those with FIB-4 >3.25? Use of mildly increased bilirubin in the definition allows inclusion of those with Gilbert's gene polymorphisms.”
 **Response: Our definition of cirrhosis included increased INR or increased bilirubin plus low platelet count AND cirrhotic looking liver (hence a combination of imaging and laboratory variables, which are recommended by the European Association for the Study of the Liver as referenced) or a combination of 3 laboratory parameters (two of coagulopathy and one of internal liver physiology). We believe there has been a confusion, and we have changed the wording for better clarification (page 6, lines 135-140). FIB4 and APRI scores have been reported to have a potential use for fibrosis assessment, but no international hepatology guideline supports them for cirrhosis diagnosis.**

4. Comment “Page 6: Only a subset of patients had testing for surface antibody after vaccination. How did these differ from the non-tested population?”

**Response: Demographics showed that those not tested for HBsAb were 63% male compared to 70% male in those tested, 14% of those not tested had cirrhosis versus 25% of those tested (this yielded a significant difference, p=0.01). Mean ages were similar between both groups: 48 y/o (CI 47-50) vs tested 54 y/o (CI 53-54), p=0.001). We have added this information to the manuscript (Page 7, line 159-163).**

5. Comment “Page 8: Comments about genetic variation not germain to your results. Indeed, with general population rates of response of >95% to 3 doses of standard vaccine, it is hard to argue that there is much effect at all due to genetic variation.”

**Response: We have placed references that studied genetic variations (references 32-33). However, we understand the comment as a practical point. We have added a comment indicating the low likelihood of this playing a role (we believe it is worth mentioning at least). Page 10, lines 237-238.**

6. Comment “Would like to sub-analysis of biopsy proven cirrhotics only before concluding that cirrhosis does not make a difference. The overall data is suspect because carefully done prospective registration trials clearly show factors like DM and dialysis clearly impact seroprotection rates. The recent report by Jackson et al (Vaccine, 2019) shows that only 65.1% of subjects receiving Engerix-B developed a response after a full series.”

**Response: Our analysis is different than that quoted by the reviewer. The reviewer quotes a study in which 65% of cirrhotics respond to HBV vaccine. We mention that among our responders there were 24% cirrhotics, these are different ways of looking at data. We have looked at the response rate among cirrhotics that got 3 doses of the vaccine, and found a response rate of 77%. This was added to the body of the study (Page 8, lines 188-193).**

7. Table 1: Confusing to read. I had to calculate the number of patients in each group. Would put N for each group
 **Response: Table 1 was modified as requested. Numbers of patients that received any number of doses was added (Table 1, Page 18).**

8. Disagree with comparisons using 2 dose recipients. Would focus on 3 dose only for reasons described above.
 **Response:** **We re-performed the analysis, assessing all responders vs non-responders with 3 or more doses (Table 2, Page 19) and Responders with 3 or more doses compared to non-responders with 3 or more doses (table 3, page 20). The “results” section, page 7, was re-written to indicate those changes and the numbers were updated.**

9. Data in Table 3 shows 3 doses matter. Would focus on that finding though it is not a surprise.

**Response: Similarly, as above, we have re-done the analysis with 3 doses and modified the table accordingly, as above.**

10. Would use multivariable statistical methods to assess impact of individual factors, not just univariate methods.

**Response: We have now included multivariate analysis to our study. On multivariate analysis we found that cirrhosis correlated with less reactivity compared to those without cirrhosis (OR 0.66, CI 0.44-0.94). In the multivariate analyses, alcohol abuse and diabetes did not show a significant correlation between HBsAb reactivity. This was added to the body of the text (Pages 9, lines 204-207).**

**Reviewer #2:**

-Comment 1: “Throughout the paper it is unclear if HBV vaccination was initiated after HCV infection occurred. Clarity on this point is needed throughout the paper”.

**Response: As the reviewer can imagine it is certainly not possible to know when patients got infected with HCV, as the great majority of infections are subclinical, we can at best estimate based on risk factor timing. To provide an estimation for the reviewer, we did assessed the percentage of first HBV vaccinations in relation to the first positive test for HCV. From a small subset of individuals (255) in which we assessed this information, we found that 177 had the first vaccination before being tested for hepatitis C. It should be understood however that the great majority of individuals expressed a remoted risk factor for HCV infection, with likely linking of risk-factor to infection at least 1-2 decades before laboratory check. This was added to the methods section, Page 6, lines 129-132.**

-Comment 2:
“Estimates of HBV and HCV prevalence utilize old estimates. The improved estimates can be found at the WHO website:
https://www.who.int/hepatitis/publications/global-hepatitis-report2017/en/”

**Response: We have added new estimates per the reviewer’s request (Page 4, lines 76-77).**

-Comment 3: “study, conducted” the comma should be removed
 **Response:** comma removed **(Page 5, line 104).**

-Comment 4: “It would read better if the single sentence first paragraph was edited into two or more sentences.”
 **Response: The paragraph was edited into 2 sentences (Page 5, lines 104-107)**

-Comment 5:” Records that were available were reviewed. Whose records are not electronically available? About how many? Note: this information doesn’t need to be added to the manuscript if the number is extremely small (e.g., <5)”.

**Response: Quote “all available” just means that all the records that we had were reviewed. We changed the paragraph per reviewers request (Page 5, line 108).**

-Comment 6: “Clinical information of the patients were collected” – consider beginning a new paragraph.

**Response: The sentence was changed to a new paragraph (Page 5, line 113).**

-Comment 7: “Hepatitis B Surface Antibody (HBsAb)” add the word “test” or when blood drawn.

**Response: We added “test” following HBsAb throughout the study.**

-Comment 8: “Differences between continuous variables were evaluated at mean, using a T-Test for statistical significance.” The t-test compares the means. This sentence needs to be rewritten. Also, if you are using a t-test then it would help to provide the means and standard deviations.

**Response: We have changed the wording of the phrase after talking to our Statistitian to: “Statistical analyses were performed using SAS version 9.4. Differences between continuous variables were evaluated at the means using a T-Test. Categorical variables were evaluated using a Chi-Square test. The multivariate regression was modeled as a logistic regression.” This is reflected on Page 6, lines 147-149.**

-Comment 9: “(n=1276) received various doses of HBV vaccination, 50% (n=646) of which were checked for HBsAb afterwards. Among those, 65% (n=418) developed HBsAb after vaccination (vaccine responders) and 35% (n=228) did not (vaccine non-responders).” This information is inconsistent with what is in Table 1. Specifically, in Table 1 there has 185 vaccine responders.

**Response: The 185 patients noted as responders in table 1 are the responders with known number and timing of doses. The 418 responders noted in the text are all patients with documented vaccination in the EMR, but not all had available timing and number of doses.**

**Only the 185 patients were included in analyses pertaining to number of doses, however the remainder of patients were included in analyses regarding clinical and virus related variables, as these were available. Notably, we also re-did all the analyses for those with known 3 or more doses in the responder arm, compared to non-responders (table 3, page 20) and that yielded similar results when compared to the analyses in table 2, page 19. This was also clarified in the results section of the study, page 8, lines 200-202.**


-Comment 10: ‘The other confusing issue is when patients received HBV vaccine. The methods suggests: that only those who were vaccinated after HCV diagnosis are studied, however this isn’t clear throughout the paper.”

**Response**: This point was address above. **We have indicated the number of individuals that received vaccination before and after testing positive to clarify this point, page 6, lines 129-132.**

-Comment 11: “-A flow chart would greatly enhance the reader’s understanding of the data. The flow chart could also clarify if the HBV vaccines started before or after HCV diagnosis. Did you exclude patients who were vaccinated for HBV before HCV diagnosis? If so, providing this information on a flow chart would help the readers.”

**Response: A flow chart was added (Page 17, figure 1).**

-Comment 12: “Tables in general would benefit from changing the percentages direction. The question of interest is: What proportion of those with risk factor X are responders?”

**Response**: **We appreciate the reviewers comment. However, our study is looking at which factors are associated with no response, which we believe is reflected in our tables. Changing the positioning of the tables would require changing also the analyses and reflective p values and we believe that would be a total different manuscript than the one we are submitting. We have however, considerably modified the tables based on the re-analysis.**

-Comment 13: “Table 1 would also benefit from the addition of a line for patients who have been vaccinated according to the guidelines.”

**Response: All patients with 3 doses were vaccinated per the guidelines. Those in the study who had interrupted timings restarted the 3 dose sequence and ended up receiving 4 or more doses.**

-Comment 14 “median age at the time of the HBsAb check was 54 years in both of the groups (IQR: 47-59 and 46-50 for responders and non-responders respectively).” This can’t be correct for non-responders.
 **Response: The age analysis was redone to identify non-responders as those who did not respond after getting at least 3 doses. Means have now changed for non-responders (Page 8, lines 177-179).**

-Comment 15: “Comparison of patients who had two HBV vaccines lacks logic. The comparisons should be for patients who were vaccinated according to guidelines. You noted a difference between 2 and 3+ doses.”
 **Response**: **Comparisons between patients with 2 doses were removed. Now the comparison is between patients who received 3 or more doses (Page 20, table 3).**

-Comment 16: “It would help if the analyses of risk factors are adjusted by age and sex. If age is put into categories then this adjusted analyses could still be conducted with PROC FREQ. This is particularly important for analysis of cirrhosis given that these individuals will be skewed toward older ages.”

**Response: We have performed and added a multivariate analysis and included age per reviewer’s request (Pages 9, lines 204-207).**

-Comment 17: “Table 3 is not helpful. It brings into the analysis patients who obtained one HBV vaccine.”
 **Response: Table 3 now modified and analysis was re-done to include patients who received 3 or more vaccines only (Page 20, table 3).**

-Comment 18: “Recommend removing “largest” study given that there are likely larger studies.”

**Response: Through our literature review, we have not encountered a study of a similar size, aside from the noted meta-analysis. However, the phrase was substituted with “one of the largest studies”, per the reviewer’s request (Page 9, line 210).**

-Comment 19: “The word “decreased” suggests a change. It may be more accurate to say “lower”.
**Response: The word “Decreased” was switched to lower, when pertaining to the response to the vaccine, throughout the study.**

-Comment 20 “In the discussion there is suggestion that patients were HBV vaccinated after HCV diagnosis. When vaccines occurred needs to be clear throughout the paper”

**Response: We have added a clarification on the percentage of patients that received vaccination before or after diagnosis. We would like to emphasize that this really does not correlate with “hepatitis C acquisition” as that is not possible to define, since most infections are subclinical. Clarification was added to the “methods”, page 6, lines 129-132.**

**Additional comments:**

-A relatively conservative estimate of HBV vaccine protectiveness was used. What if that cutoff were increased to 100?

**Response: Our cut off (a titer of 12 mIU/mL) is above the recommended cut off of 10 mIU/mL, deemed to be protective against hepatitis B (West DJ, Calandra GB. Vaccine induced immunologic memory for hepatitis B surface antigen: implications for policy on booster vaccination. Vaccine. 1996;14(11):1019‐1027.), which is also the cut off recommended by the CDC.**

-Were the sociodemographic or clinical characteristics of those that were checked for anti-HBs different from those that were not checked? (Regardless of the findings)

**Response: We assessed the characteristics of those who were tested to document immunity and those who were not, patients who were not tested were younger, both groups were predominantly male, this data was added (Page 7, Lines 159-163).**

-What was the HIV status of the individuals in the responder versus non-responder groups?

**Response: Because of the low number of individuals with HIV (<2%) in the population, these individuals were removed from the study.**

- How were alcohol and intravenous drug use defined? Self-report? Ever / never?

**Response**: **History of alcohol abuse, was obtained from the chart, by assessing for documentation of use compatible with abuse or for a diagnosis of alcohol abuse, related to recurrent alcohol related presentations and positive toxicology in those who did not self-report heavy use. To ensure the patients had active alcohol abuse at the time of vaccination, only patients who were noted to have ongoing abuse in the hepatology clinic were defined as abusers for the purpose of the study.**

**IV drug use was obtained from EMR charts and problem lists that could have been a consequence of previous positive toxicology or relied on self-report. Patients were considered to have IV drug abuse if the evaluating provider noted a history of active or recent drug use on presentation to the hepatology clinic.**
