# Supplementary material for: Response to hepatitis B virus vaccination in individuals with chronic hepatitis C virus infection
Source: PLoS One. 2020 Aug 26;15(8):e0237398. doi: 10.1371/journal.pone.0237398 (PMC7449383; doi:10.1371/journal.pone.0237398)
Supplement: Attachment — Submitted filename: Response to Reviewers.docx [file pone.0237398.s003.docx]

The authors appreciate the reviewer’s thoughtful and insightful comments, as well as the time and effort spent.

1. Abstract: The authors conclude that HCV infection impairs HBV vaccine response with cirrhosis being the only identifiable risk factor. This conclusion is not supported by the data presented. There is no evidence that HCV specifically impairs HBV vaccine response. Indeed, the statement actually says that cirrhosis is the factor that impairs response (among those with HCV). This distinction is important.

**Response: We appreciate the reviewer’s comment. Our data shows a 79% HBV vaccine response, that is much lower than the >90% reported in the general population in multiple studies, and a 50% response rate in the population who received 3 or more doses. We have changed the comment in the abstract to clarify (page 3, line 66). To further clarify this to the readers, we have added the response rates in non-cirrhotic HCV patients in our cohort, which is also well below the rate reported in the literature, to the results section (page 8, lines 188-191).**

2. Page 7: I remain confused about the numbers and calculations. The rate of response in those that received 3 doses was 42%. Then the authors note that 77% of those that received 3 or more doses had a response but in parentheses it says (32/139). This is a 23% response rate.

**Response: We apologize for the confusion regarding the second portion of the comment, we made a typo (32 were non responders, but 107 were, accounting for a response rate of 77% as indicated). We have fixed this issue in the manuscript (page 8, line 187). We have to emphasize however, that as indicated in that line, this is the response rate in cirrhotics who received 3 or more doses. When assessing the entire population who received 3 or more doses, is detailed in table 3, page 20, response rate is 50% (equal number of patients in each arm).
* Clarification: The “42%” noted in table 1, is not the response rate, but rather, the proportion of responders, who sero-converted following 3 doses of the vaccine, as is indicated in the column title.**

3. The overall response rate in non-cirrhotic patients who completed 3 or more doses is not explicitly stated. However, the overall rate of response among those who received 3 or more doses was 79.5%.

**Response: Thank you for the comment. As we indicated above, we have added the lines 188-191, in page 8, to clarify the response rate in non-cirrhotics only, which as indicated above, is significantly lower than the response rate in the general population.**

4. In univariate analysis there was no difference in response related to cirrhosis with the proportions of cirrhotics among responders and non-responders essentially equal (p= 0.6). To then select a subset of characteristics and find significance for cirrhosis is a bit of statistical gamesmanship.
 **Response: We appreciate the reviewer’s comment. However, we carefully chose the characteristics (Age, gender, alcohol abuse, and DM) as each of these has been reported to be associated with decreased HBV vaccine response in the literature. We have added a comment to clarify this (Page 9, line 203). Seeing as the proportion of Cirrhosis was higher in the non-responders’ arm, but no statistically, a concern was that those characteristics may have been confounders that limited the univariate analysis. A multivariate analysis accounting for possible confounders was also suggested by reviewer 2 at the time of the initial submission.**

5. Page 7: After stating that 418/525 (79.6%) have a vaccine response, the next sentence says "Out of all who receive 3 or more doses only 50% develop HBsAb.

**Response: Our initial statement says “various doses” indicating that we are including patients that responded even with 2 doses of the vaccine. The statement includes those that received all 3 doses. We have changed “various” to “any number” of HBV vaccine doses to make that more clear (page 7, line 161-162).**
